# Supplementary material for: Regulatory Cross Talk Between SARS-CoV-2 Receptor Binding and Replication Machinery in the Human Host
Source: Front Physiol. 2020 Jun 30;11:802. doi: 10.3389/fphys.2020.00802 (PMC7338756; doi:10.3389/fphys.2020.00802)
Supplement: Supplementary file 6 [file Presentation_1.PPTX]

## Slide 1
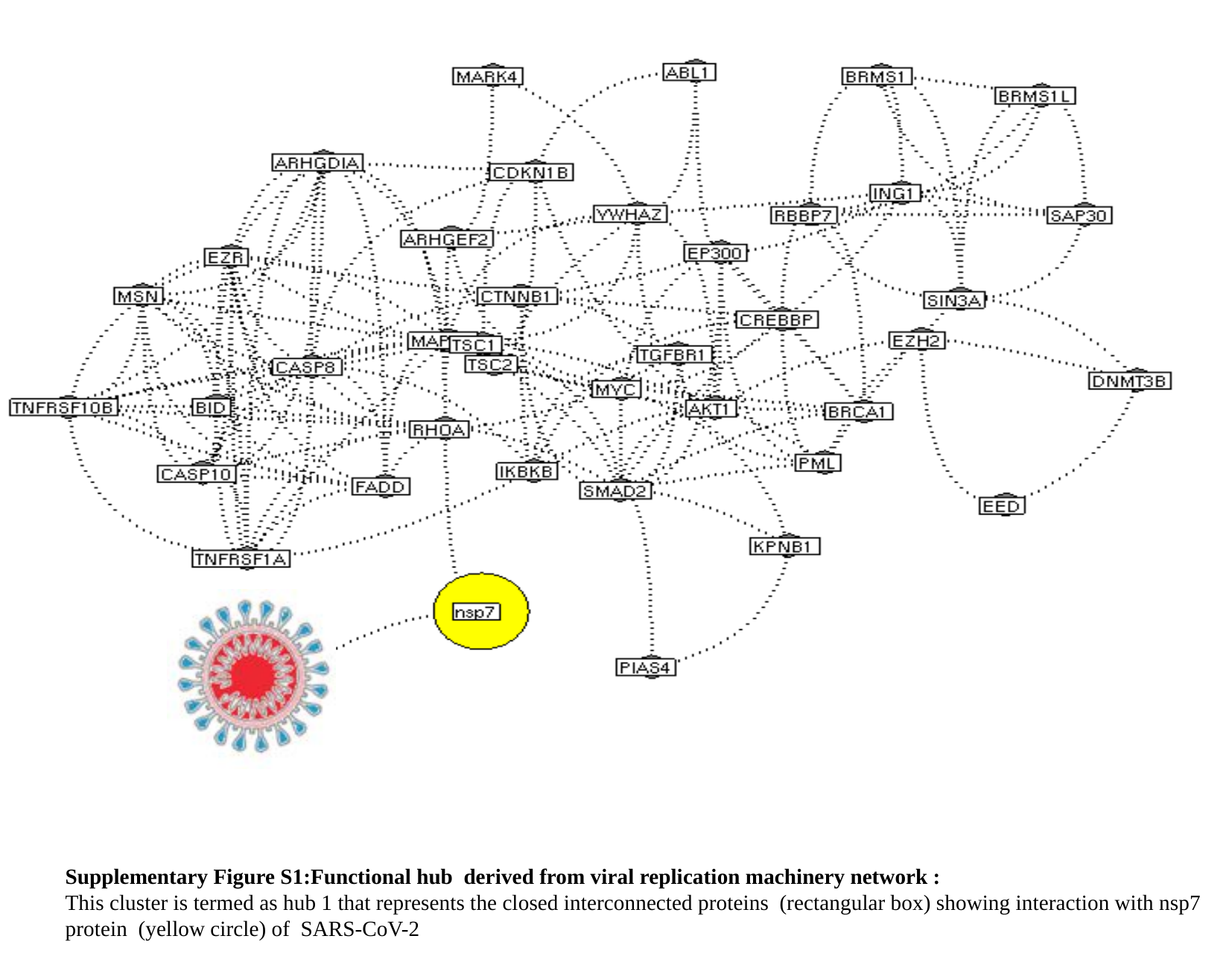

# Supplementary Figure S1:Functional hub derived from viral replication machinery network : This cluster is termed as hub 1 that represents the closed interconnected proteins (rectangular box) showing interaction with nsp7 protein (yellow circle) of SARS-CoV-2

## Slide 2
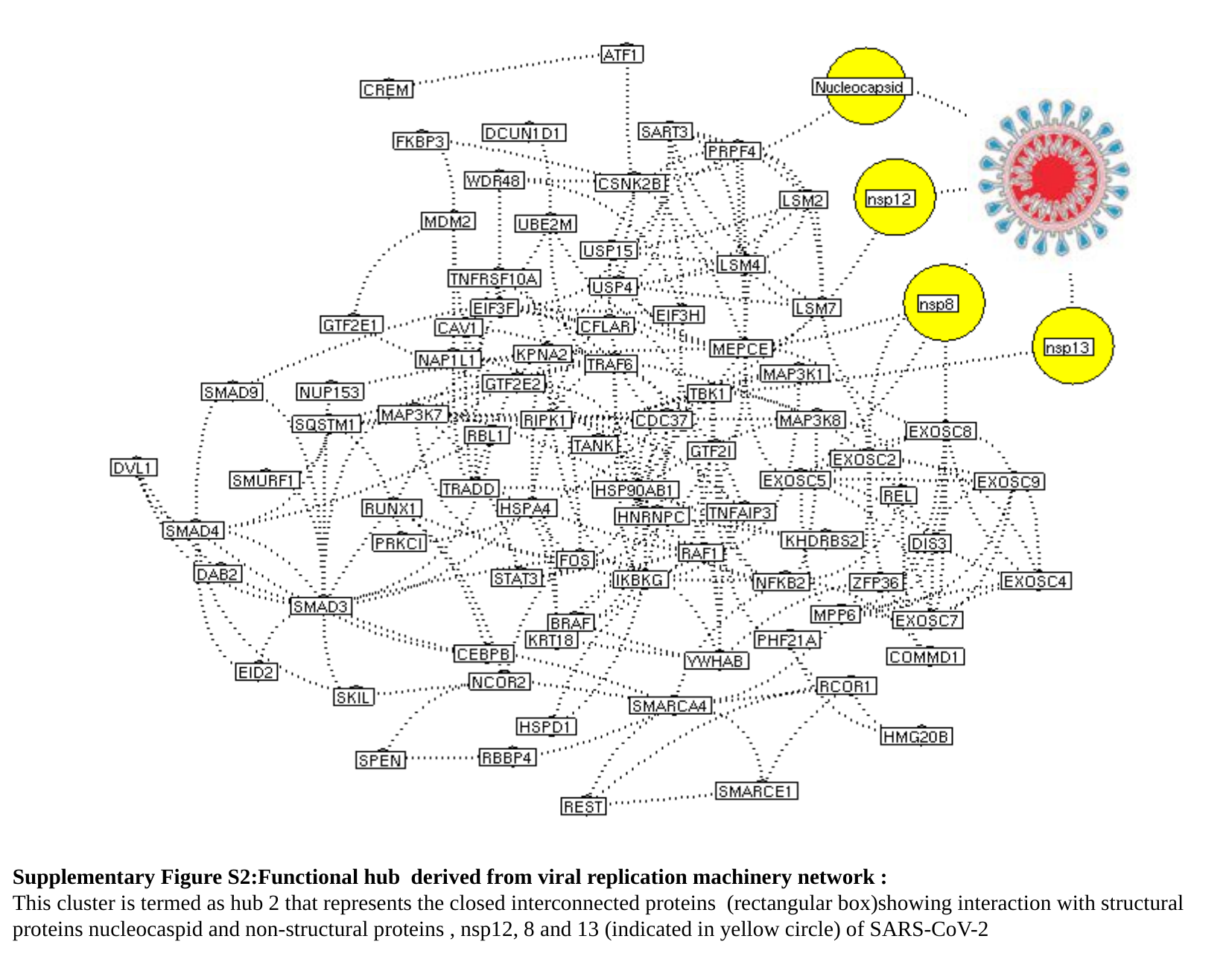

# Supplementary Figure S2:Functional hub derived from viral replication machinery network : This cluster is termed as hub 2 that represents the closed interconnected proteins (rectangular box)showing interaction with structural proteins nucleocaspid and non-structural proteins , nsp12, 8 and 13 (indicated in yellow circle) of SARS-CoV-2

## Slide 3
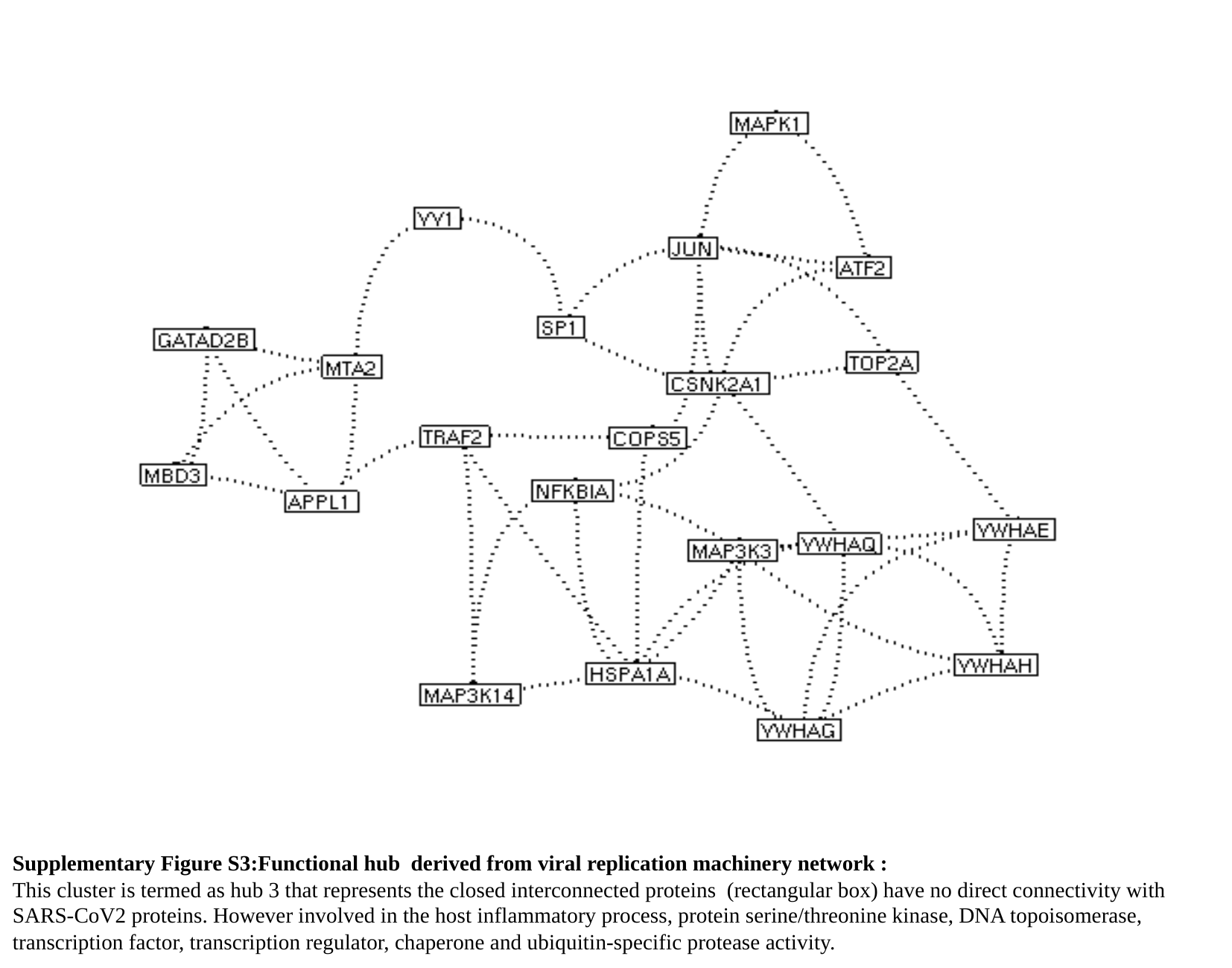

# Supplementary Figure S3:Functional hub derived from viral replication machinery network : This cluster is termed as hub 3 that represents the closed interconnected proteins (rectangular box) have no direct connectivity with SARS-CoV2 proteins. However involved in the host inflammatory process, protein serine/threonine kinase, DNA topoisomerase, transcription factor, transcription regulator, chaperone and ubiquitin-specific protease activity.

## Slide 4
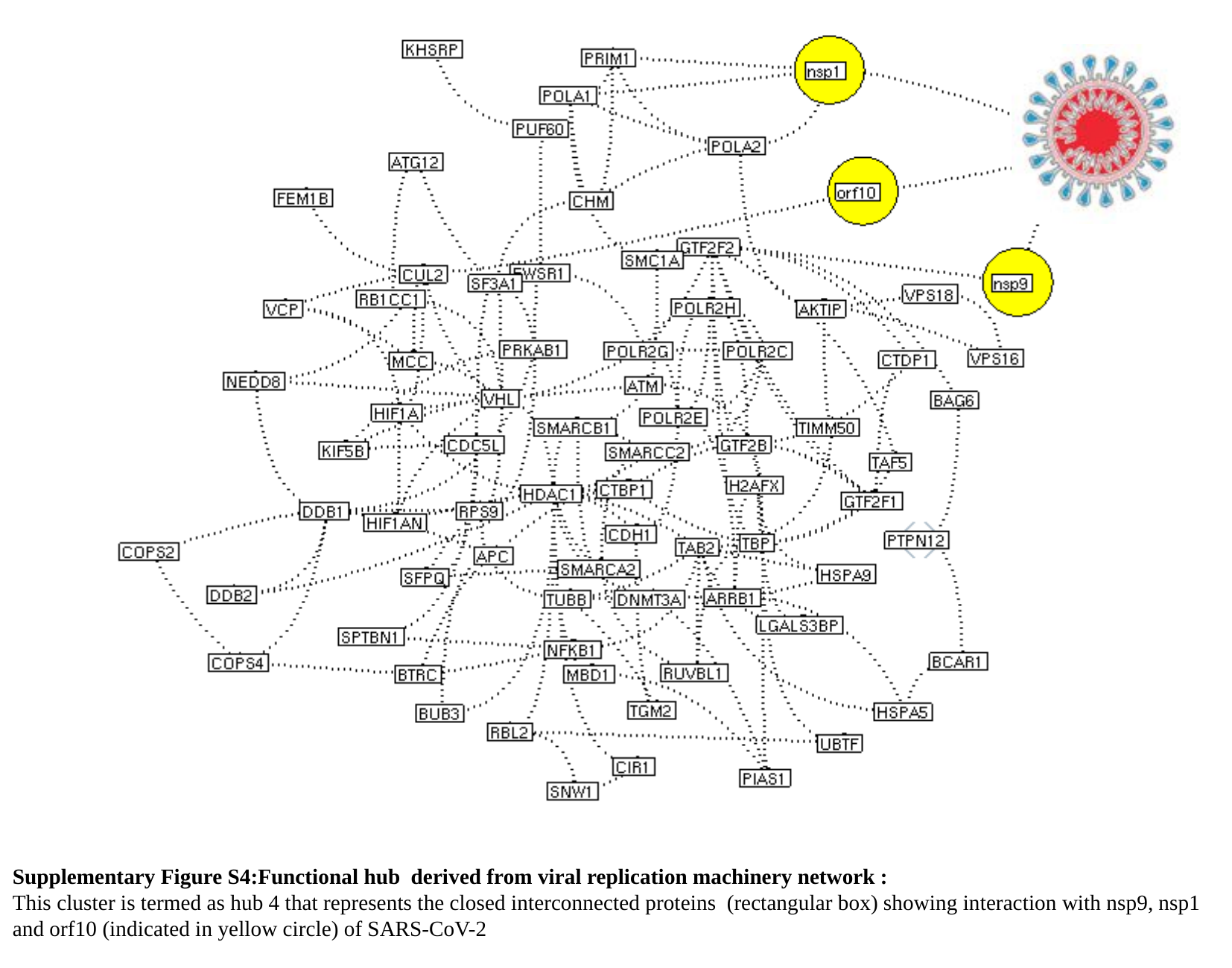

# Supplementary Figure S4:Functional hub derived from viral replication machinery network : This cluster is termed as hub 4 that represents the closed interconnected proteins (rectangular box) showing interaction with nsp9, nsp1 and orf10 (indicated in yellow circle) of SARS-CoV-2

## Slide 5
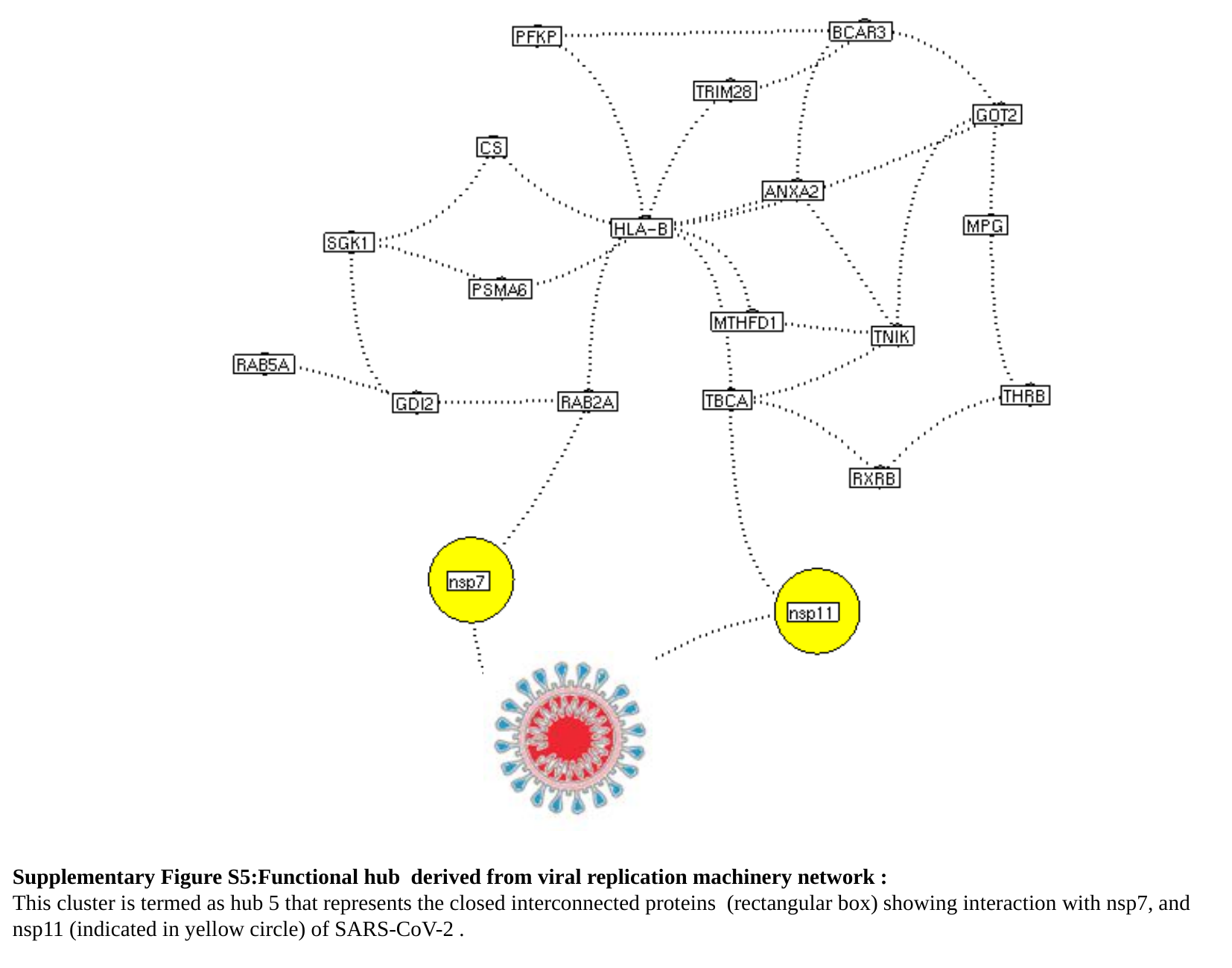

# Supplementary Figure S5:Functional hub derived from viral replication machinery network : This cluster is termed as hub 5 that represents the closed interconnected proteins (rectangular box) showing interaction with nsp7, and nsp11 (indicated in yellow circle) of SARS-CoV-2 .

## Slide 6
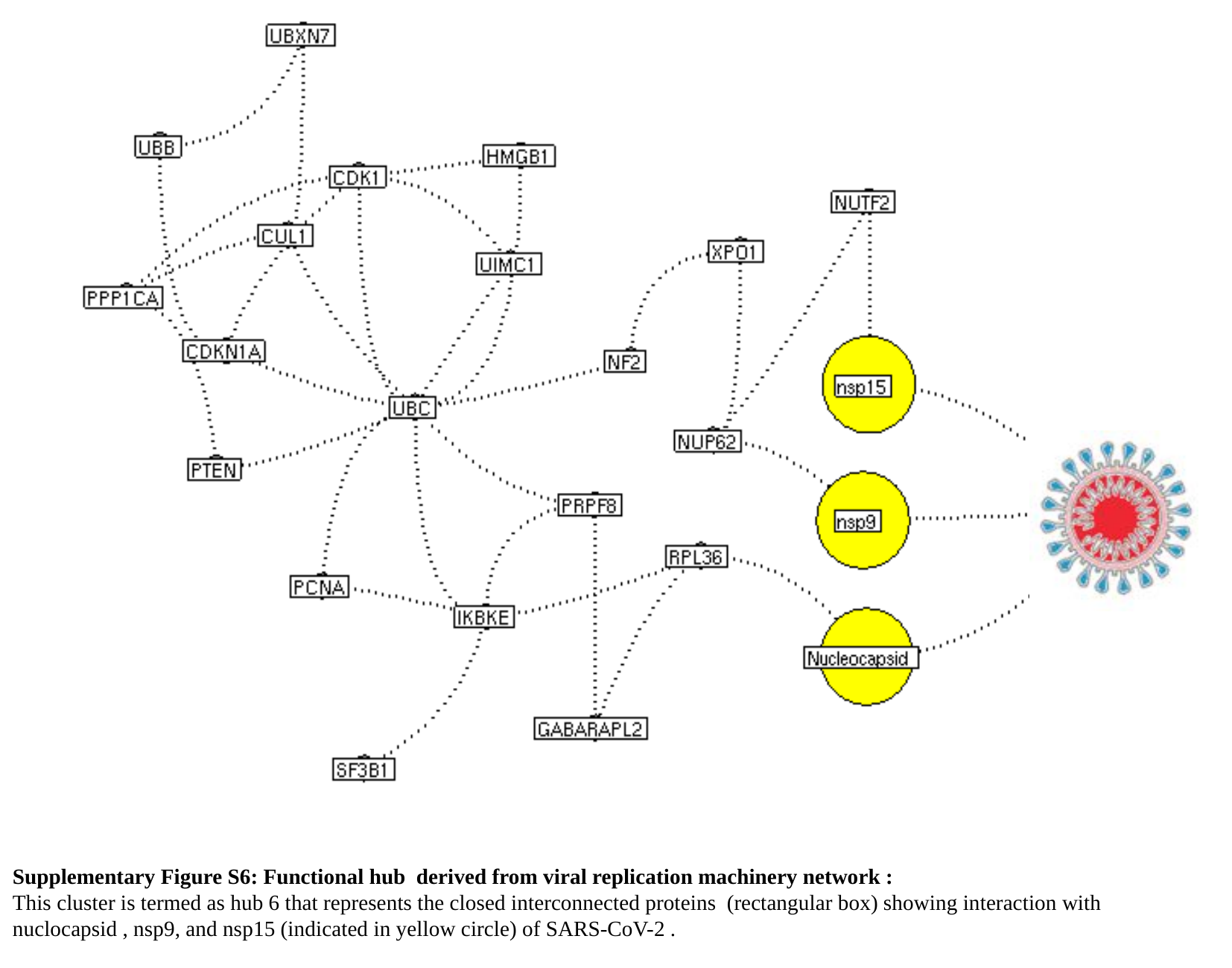

# Supplementary Figure S6: Functional hub derived from viral replication machinery network : This cluster is termed as hub 6 that represents the closed interconnected proteins (rectangular box) showing interaction with nuclocapsid , nsp9, and nsp15 (indicated in yellow circle) of SARS-CoV-2 .

## Slide 7
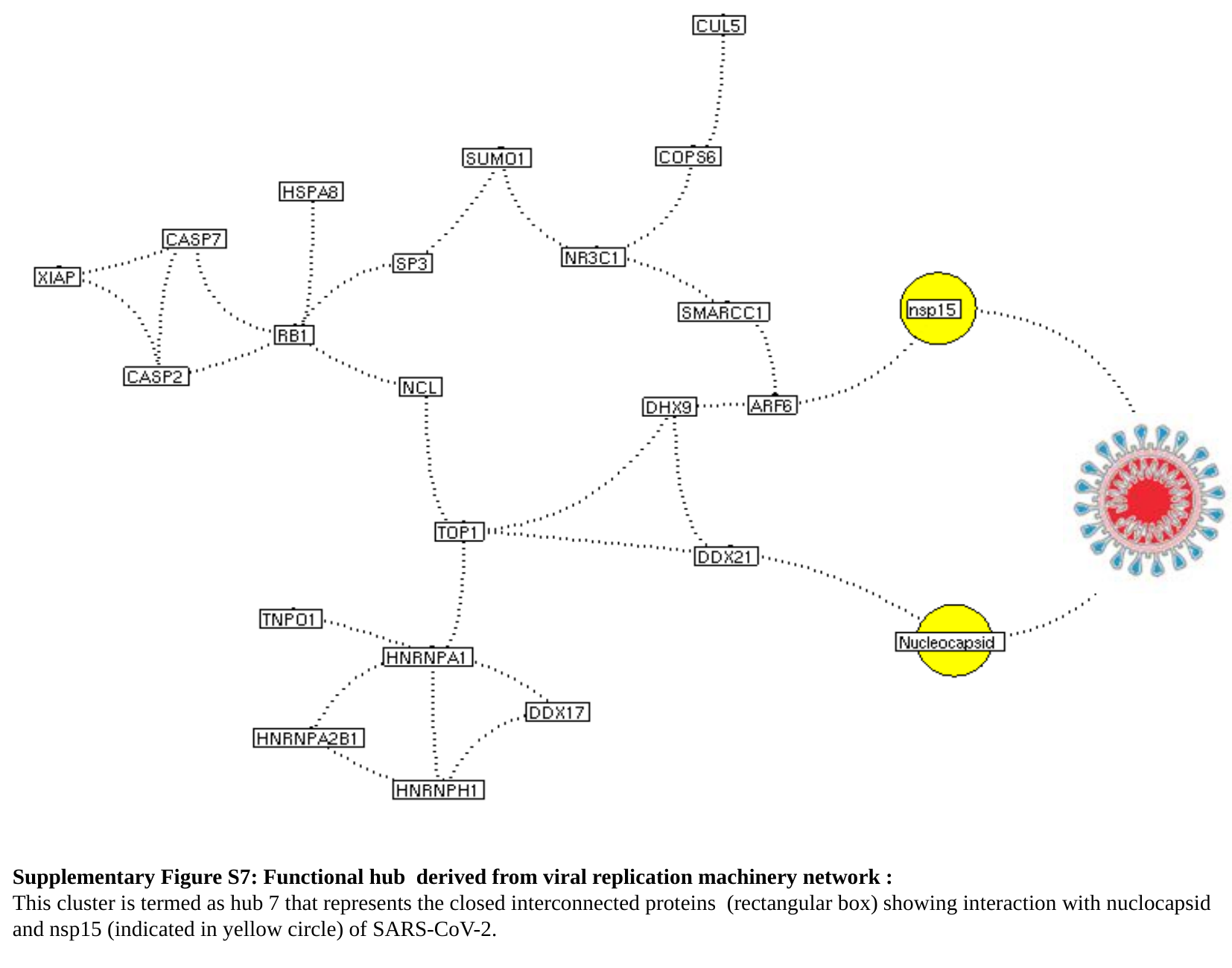

# Supplementary Figure S7: Functional hub derived from viral replication machinery network : This cluster is termed as hub 7 that represents the closed interconnected proteins (rectangular box) showing interaction with nuclocapsid and nsp15 (indicated in yellow circle) of SARS-CoV-2.

## Slide 8
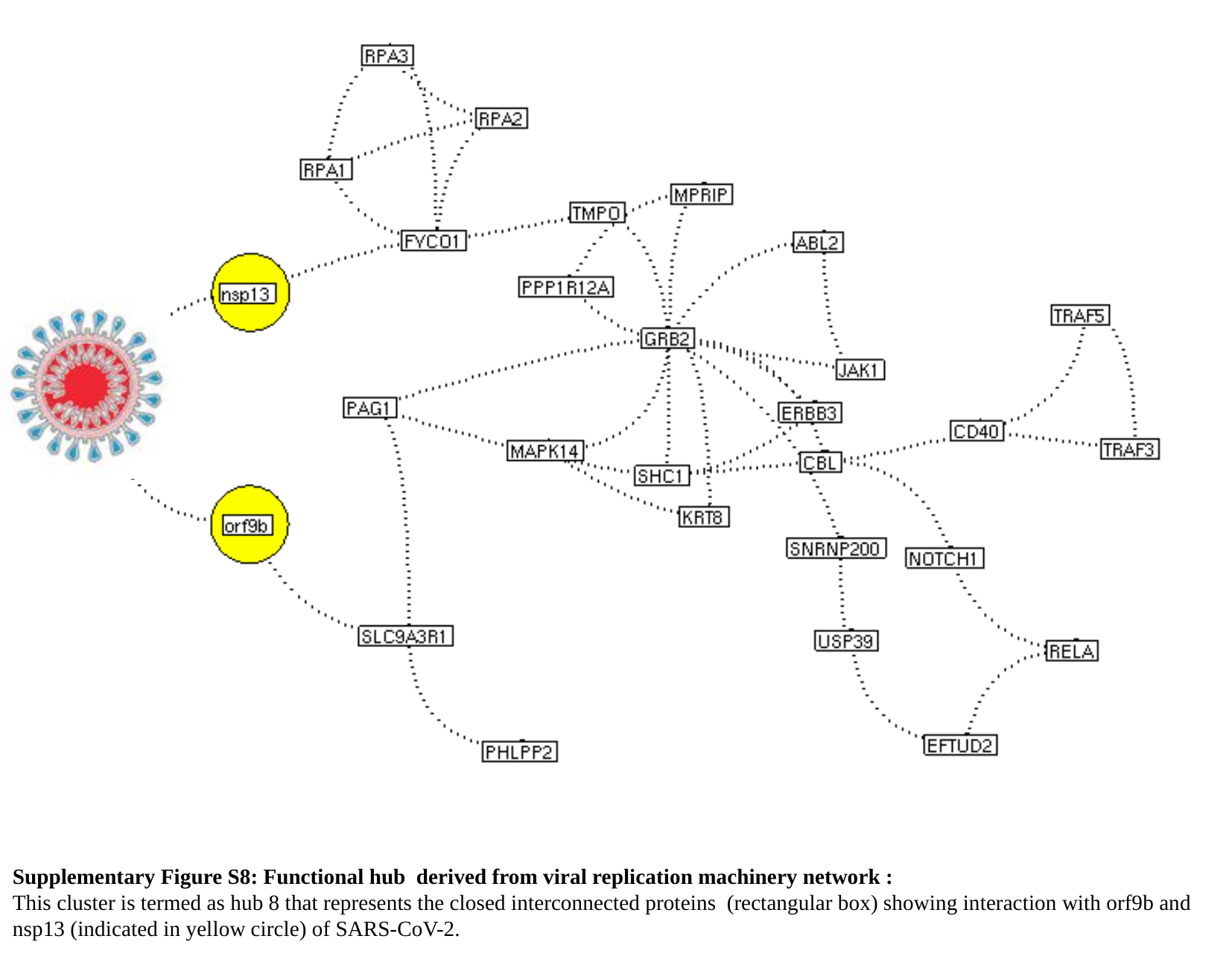

# Supplementary Figure S8: Functional hub derived from viral replication machinery network : This cluster is termed as hub 8 that represents the closed interconnected proteins (rectangular box) showing interaction with orf9b and nsp13 (indicated in yellow circle) of SARS-CoV-2.

## Slide 9
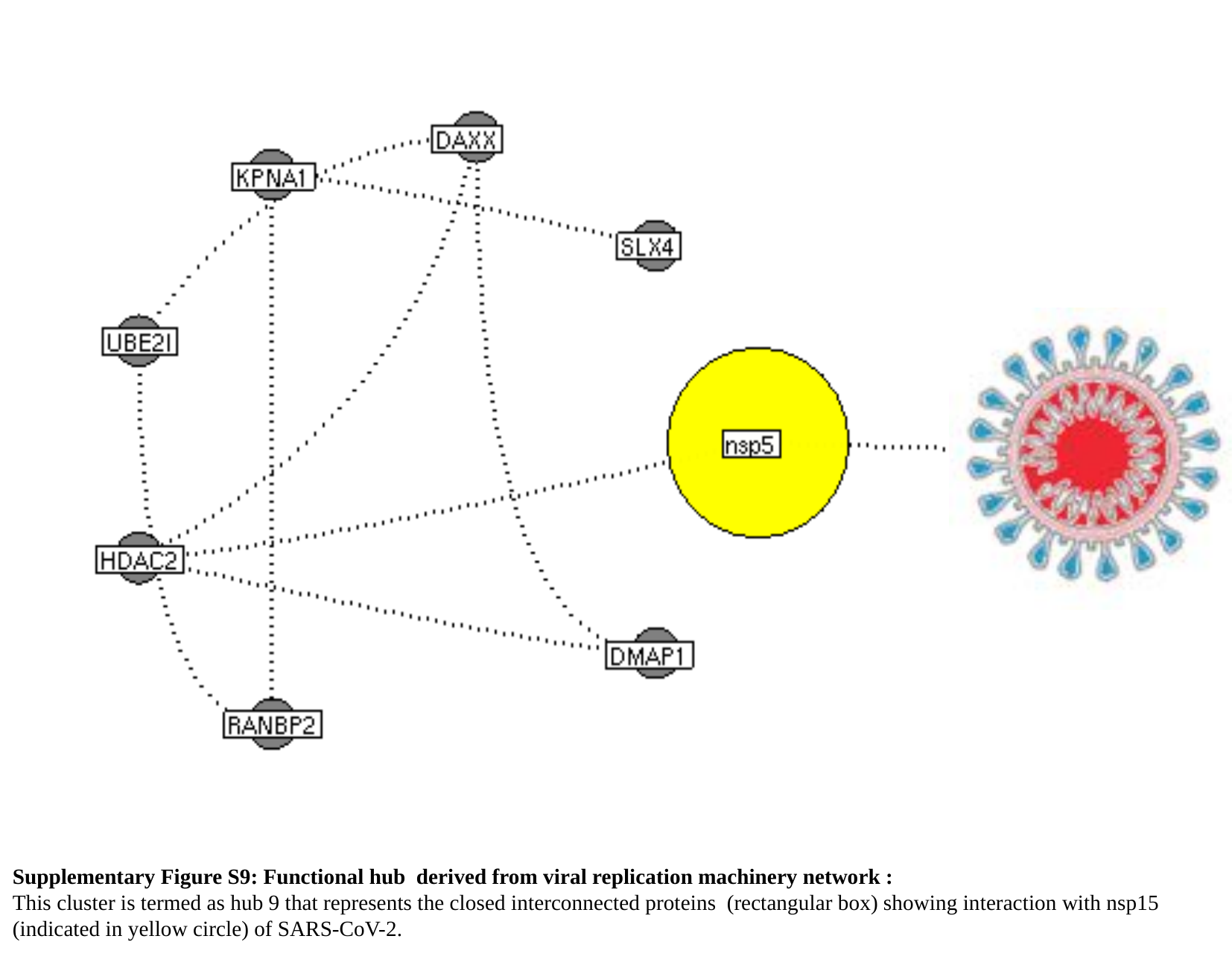

# Supplementary Figure S9: Functional hub derived from viral replication machinery network : This cluster is termed as hub 9 that represents the closed interconnected proteins (rectangular box) showing interaction with nsp15 (indicated in yellow circle) of SARS-CoV-2.

## Slide 10
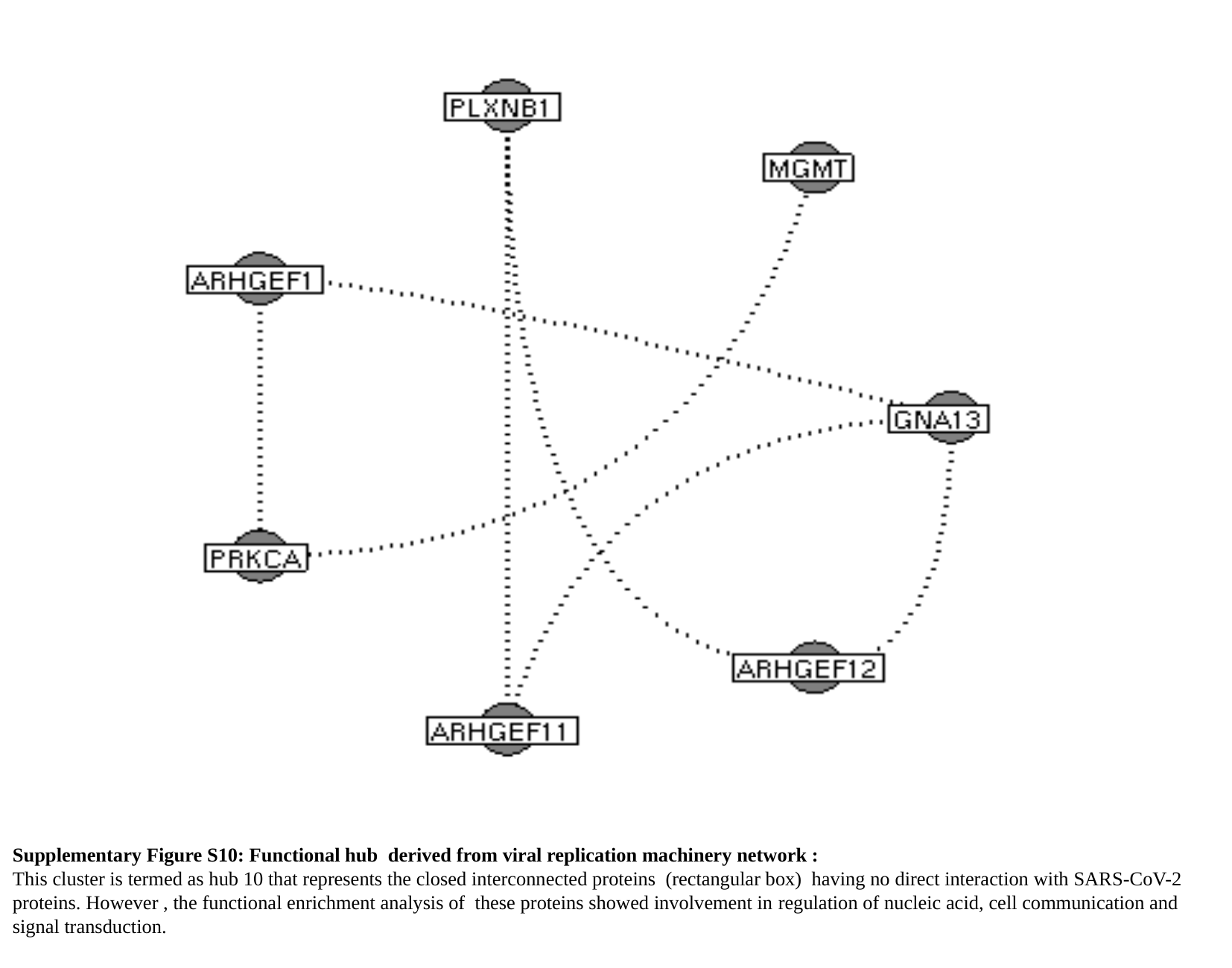

# Supplementary Figure S10: Functional hub derived from viral replication machinery network : This cluster is termed as hub 10 that represents the closed interconnected proteins (rectangular box) having no direct interaction with SARS-CoV-2 proteins. However , the functional enrichment analysis of these proteins showed involvement in regulation of nucleic acid, cell communication and signal transduction.

## Slide 11
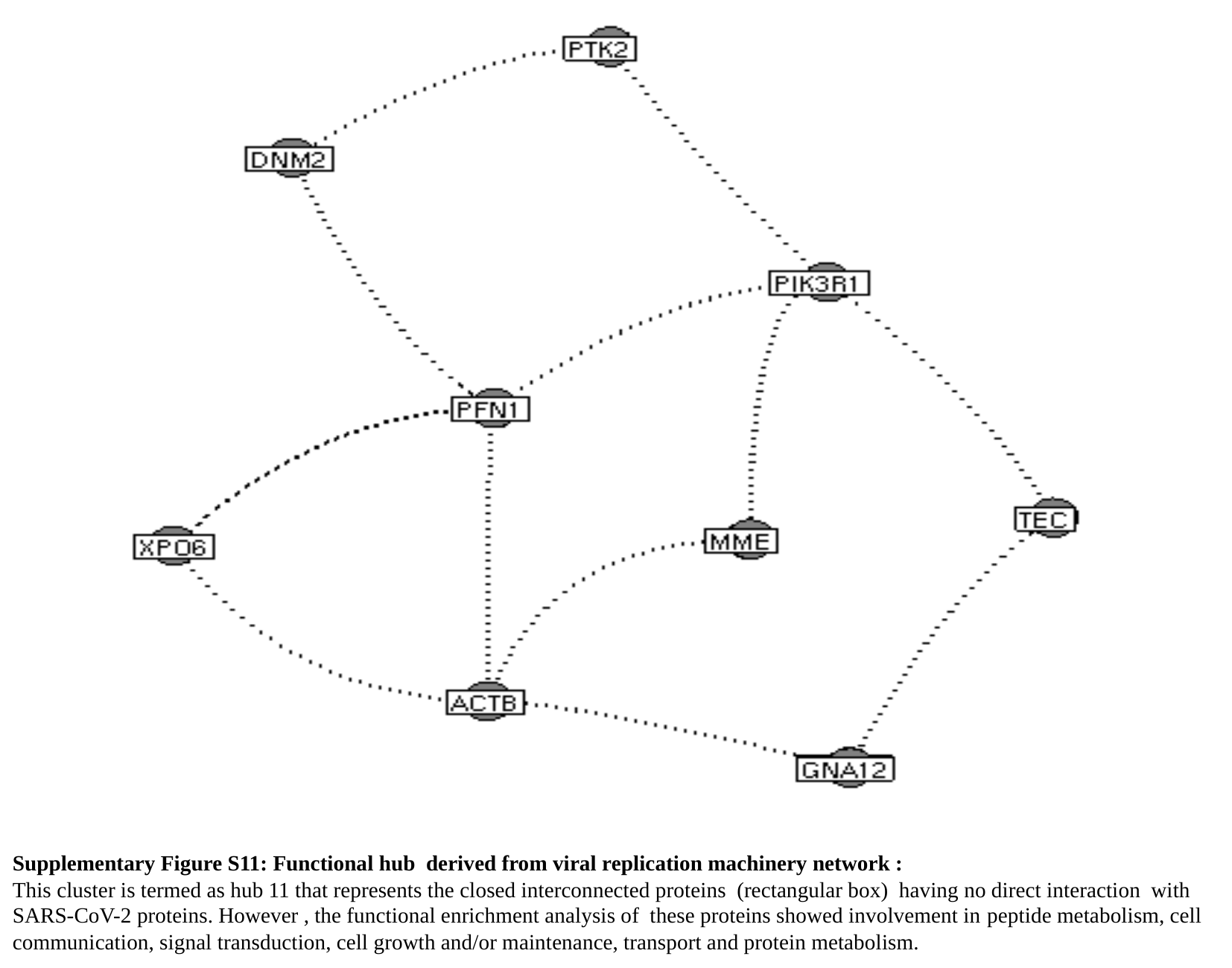

# Supplementary Figure S11: Functional hub derived from viral replication machinery network : This cluster is termed as hub 11 that represents the closed interconnected proteins (rectangular box) having no direct interaction with SARS-CoV-2 proteins. However , the functional enrichment analysis of these proteins showed involvement in peptide metabolism, cell communication, signal transduction, cell growth and/or maintenance, transport and protein metabolism.
